# Rapid and portable bunyavirus SFTSV RNA testing utilizing catalytic hairpin assembly coupled with lateral flow immunoassay

**DOI:** 10.1128/spectrum.02144-23

**Published:** 2023-09-08

**Authors:** Lin Chen, Mengyin Ma, Mingyuan Zou, Liwei Zhao, Mingrong Ou, Yu Geng, Chuang Li, Han Shen, Yuxin Chen

**Affiliations:** 1 Department of Laboratory Medicine, Nanjing Drum Tower Hospital Clinical College of Nanjing Medical University, Nanjing, Jiangsu, China; 2 Department of Thoracic Surgery, the First Affiliated Hospital of Guangzhou Medical University, Guangzhou, China; 3 Department of Laboratory Medicine, Nanjing Drum Tower Hospital Clinical College of Traditional Chinese and Western Medicine, Nanjing University of Chinese Medicine, Nanjing, Jiangsu, China; 4 Department of Infectious Diseases, Nanjing Drum Tower Hospital Clinical College of Traditional Chinese and Western Medicine, Nanjing University of Chinese Medicine, Nanjing, Jiangsu, China; 5 Institute of Viruses and Infectious Diseases, Nanjing University, Nanjing, Jiangsu, China; Houston Methodist Hospital, Houston, Texas, USA

**Keywords:** SFTSV, catalytic hairpin assembly, lateral flow immunoassay, POCT

## Abstract

Severe fever with thrombocytopenia syndrome (SFTS) is a prevalent, life-threatening, emergent infectious disease. Currently, reverse transcription-polymerase chain reaction is the gold standard for diagnosing SFTS virus (SFTSV) infection, which requires sophisticated equipment and professional personnel that are frequently unavailable in most SFTS endemic rural areas. Here, we reported a simple, rapid nucleic acid amplification system that combined the catalytic hairpin assembly (CHA) with a lateral flow immunoassay (LFIA) strip-based detection method for SFTSV detection. The detection of SFTSV RNA could be realized by generation of H1-H2 hybrid duplexes labeled with biotin and digoxin, which subsequently added to the LFIA test strips containing streptavidin conjugated with Alexa Fluor 647 as well as anti-digoxin antibodies. Our CHA-based LFIA assay offered high amplification efficiency and specificity with a detection limit of 1 aM. Crucially, this method enabled stable detection of 500 copies/mL of SFTSV within 30 min using clinical serum samples. Therefore, our CHA-based LFIA approach provided a potential useful tool to facilitate early and precise diagnosis of SFTS patients in poorly resourced SFTS endemic areas.

**Importance:**

Severe fever with thrombocytopenia syndrome (SFTS) is an emerging and potentially fatal infectious disease prevalent in China. Here we report a simple, rapid nucleic acid amplification system, the catalytic hairpin assembly (CHA) in conjunction with a lateral flow immunoassay (LFIA) strip-based detection method for SFTS virus detection, which demonstrated high amplification efficiency and specificity with limit of detection of 1 aM. Most importantly, we also validate our CHA-based LFIA assay using the clinical serum samples, which was fully compatible with reverse transcription-PCR results. Therefore, our strategy provides a potential useful tool to facilitate early and precise diagnosis of SFTS patients especially in poorly resourced SFTS endemic areas.

## INTRODUCTION

*Dabie bandavirus*, known as severe fever with thrombocytopenia syndrome virus (SFTSV) was an emerging tick-borne virus belonging to genus *Phlebovirus* in the Bunyaviridae family (1). SFTSV infection is a highly fatal emerging infectious disease, termed as SFTS. SFTS has a high fatality rate of up to 30%, which was correlated with age and viral RNA load (2, 3). The major clinical manifestation of SFTS includes fever, hematologic abnormalities, gastrointestinal symptoms, as well as non-specific clinical symptoms such as anorexia, fatigue, nausea, abdominal pain, vomiting, etc. (4, 5).

Since the first discovery of SFTSV in 2009, SFTS cases were reported in central and eastern regions of China (1, 6), South Korea, Japan, Pakistan, and Vietnam (7 – 11). It is generally accepted that SFTSV is majorly transmitted via tick bites (12, 13), but also person-to-person transmitted via contacting the body fluids of SFTS patients (14, 15). Given the high mortality and wide spread of SFTS, there was an urgent need for a sensitive, accurate, and simple detection method for early screening and diagnosis of SFTSV.

A variety of methods have been developed to diagnose SFTSV infection, including virus isolation, the detection of virus RNA, and SFTSV-related antibodies. Direct evidence of SFTSV infection was virus isolation from virus carriers; however, this procedure was time-consuming and required high biosafety equipment (16). The nucleocapsid (N) protein in the viral structure of SFTSV was highly immunogenic, which was frequently used in serological assays to detect specific SFTSV antibodies. The routine approach to determine SFTSV infection in clinical laboratories was the detection of RNA of SFTSV by reverse transcription-polymerase chain reaction (RT-PCR), which requires the professional laboratory staffs to perform the testing. Meanwhile, several assays based on the detection of viral nucleic acid have been developed for the diagnosis of SFTS, including the clustered regularly interspaced short palindromic repeat (CRISPR) and reverse transcription loop-mediated isothermal amplification (17, 18). Nevertheless, these methods were labor intensive and could not be quickly applied during clinical practice. Therefore, it was imperative to establish a novel, simple, portable SFTSV nucleic acid assay, which could be easily applied in rural areas and field investigation.

Catalytic hairpin assembly (CHA) is a high-efficient and enzyme-free method of isothermal amplification that allows for the tracking of trace amounts of nucleic acid sequences through amplifying signals (19). Compared with conventional RT-PCR, CHA demonstrated high amplification efficiency in fast manner. Furthermore, CHA could be coupled with other techniques, such as CRISPR-Cas9, DNAzyme, hybridization chain reaction, to improve the sensitivity and specificity, and is extensively used for the detection of microRNA, DNA, metal ions, protein, and tumor exosome (20 – 23). In addition, combining analytes with a variety of sensing modalities, such as fluorescence and electrochemical signals, would simplify the measurement (24, 25). Due to the advantage of fast readout and simple operation, there has been a growing demand for lateral flow immunoassay (LFIA) for disease diagnosis (26). The conventional LFIA could only be used in qualitative test for primary screening, meanwhile quantitative immunoassay could overcome the above difficulties by using label particle, achieving highly sensitive quantitative detection (27, 28).

In this study, a novel SFTSV RNA detection strategy involving target-activated CHA coupled with LFIA was developed, which provided a rapid, simplified, and sensitive nucleic acid amplification system and could efficiently monitor fluorescence signal via an LFIA strip. With the advantage of this newly established SFTSV detection method, it could provide a novel tool to promptly identify SFTSV infection and ultimately provide the appropriate treatment for SFTS patients.

## MATERIALS AND METHODS

### Probe design and oligonucleotide synthesis

The genome sequence of SFTSV was obtained from NCBI (https://www.ncbi.nlm.nih.gov/) and then subjected to ClustalW (https://www.genome.jp/tools-bin/clustalw) to select highly conserved target regions (21–50 bp) via sequence alignment. Six pairs of DNA hairpins were subsequently designed by the NUPACK software program (www.nupack.org). The NUPACK was applied to examine the secondary structures of these DNA hairpins at different temperatures (Fig. S1). The DNA and RNA oligonucleotides (Table 1) were synthesized and purified by high-performance liquid chromatography by Sangon Biotech Company, Ltd (Shanghai, China).

**TABLE 1:** List of primers and target RNA oligonucleotides^*a*^

Name	Sequence (5′−3′)
A-H1	CTTGTCCTCTTCCATCTCCACGGACAAGGAAAAAGGTGGAGATGGAAGA
A-H2	TCTCCACCTTTTTCCTTGTCCGTGGAGATGGAAGAGGACAAGGAAAAAG
A-T	GUGGAGAUGGAAGAGGACAAG
B-H1	CTGTTGACTCCTCTTTGTTCTTCAACAGTCACCCCAGAACAAAGAGGAG
B-H2	TTGTTCTGGGGTGACTGTTGAAGAACAAAGAGGAGTCAACAGTCACCCC
B-T	AGAACAAAGAGGAGUCAACAG
C-H1	GTTGACTCCTCTTTGTTCTTGAGTCAACAAAAAAGCAAGAACAAAGAGG
C-H2	GTTCTTGCTTTTTTGTTGACTCAAGAACAAAGAGGAGTCAACAAAAAAG
C-T	CAAGAACAAAGAGGAGUCAAC
D-H1	TTATACATTTCTCCGAGGGCATGTATAAGTAAGGATGCCCTCGGAGAAA
D-H2	GAGGGCATCCTTACTTATACATGCCCTCGGAGAAATGTATAAGTAAGGA
D-T	UGCCCUCGGAGAAAUGUAUAA
E-H1	CTCTTCCATCTCCACAATCTTCTTTGGAAGAGGGAGGGAAGAAGATTGTGGAGA
E-H2	ATCTTCTTCCCTCCCTCTTCCAAAGAAGATTGTGGAGATGGAAGAGGGAGGG
E-T	AAGAAGAUUGUGGAGAUGGAAGAG
F-H1	CGAGGGCACAGGACTTCTTATATGCCCTCGGAGAAATGTATAAGAAGTCCTG
F-H2	TTCTTATACATTTCTCCGAGGGCATATAAGAAGTCCTGTGCCCTCGGAGAAATG
F-T	UGCCCUCGGAGAAAUGUAUAAGAA

^*a*^ H1 and H2 are the designed hairpin probes. Digoxin and biotin were labeled at the 5′ end of hairpin and T is the targeting sequence. A–F are the six different groups.

H1 and H2 hairpin fragments and SFTSV RNA fragments were dissolved in TNaK^+^Mg^2+^ buffer (20 × 10^3^ M Tris pH 7. 5; 140 × 10^3^ M NaCl; 5 × 10^3^ M KCl; 5 mM MgCl_2_，pH 7.96）to achieve a final concentration of 10 µM and then stored at 4°C (29). Before experiments, the H1 and H2 hairpin probes were denaturized at 95°C for 10 min and annealed by being placed at 4°C for 1 h to fully form the hairpin structure.

### CHA fluorescence analysis

The feasibility of the CHA assay to detect SFTSV RNA was evaluated. carboxyl fluorescein (FAM) fluorescence and black hole quencher (BHQ-1) quencher group were labeled at the 5′ and 3′ ends of H2, respectively. Six different reactions (H1, H2, T, H1 + H2, H1 + T, and H1 + H2 + T) were set up and the final concentration of each probe was 1 µM. The fluorescence values at 37°C from 0 min to 40 min were recorded every 30 s by real-time fluorescence analysis (CFX96 C1000 Thermal Cycler, BioRad).

To optimize experimental condition, we investigated the concentration ratio, temperature, and reaction time of CHA assay. First, to determine the optimal ratio of H1:H2 concentration, the target chain and probe H1 or H2 concentration were set at 1 µM, while the concentration of the alternative probe was adjusted to 1 µM, 2 µM, 3 µM, and 4 µM. Probes and targeted sequence were mixed in the tubes at 37°C for 40 min. Next, the optimal temperatures were determined under the condition of the optimal probe concentration ratio. The CHA reaction mixture was performed for 40 min at seven different temperatures: 25°C, 30°C, 35°C, 37°C, 40°C, 45°C, and 50°C. The real-time fluorescence values were analyzed to identify the ideal reaction temperature. Finally, to identify the optimal reaction time, the assay duration from 5 min to 90 min was also analyzed based on the fluorescence values.

### Agarose gel electrophoresis

The CHA assay was also validated by agarose gel electrophoresis (AGE). The substrate purity, presence of an unspecific reaction, catalytic production, and the conversion efficiency were determined based on the fragment length and brightness values of the bands (30).

The final concentration of each probe was 1 µM. For each probe set, six reactions (H1, H2, T, H1 + H2, H1 + T, and H1 + H2 + T) were performed simultaneously in a total volume of 30 µL at 37°C for 30 min. Each sample was then added to 6 µL of 6× loading buffer. Ten microliters of the product was loaded in each well of the native agarose gel. The gels were photographed using Tanon (Shanghai, China) 2,500 UV.

### Development of CHA-based lateral flow immunoassay

Lateral flow immunoassay strips consisted of a sample pad, conjugation pad, nitrocellulose membrane with a test (T) and control (C) line, and an absorbent pad. The conjugation pad was coated with polyethylene (PE) nanoparticles that were double-tagged with streptavidin and Alexa Fluor 647. The test line was prepared with anti-digoxin antibodies, while biotin was adopted to generate the control line.

Digoxigenin-biotin bi-labeled H1-H2 hybrids, which were the amplification products to be measured, were applied 100 µL to the sample pad. Then, the fluid flows to the conjugation pad. The H1-H2 duplex reacted with double-labeled PE nanoparticles to generate H1-H2 duplex-PE nanoparticle complexes through biotin-streptavidin interactions. These complexes then proceeded along the band until they reached the test line, where they were attached to the anti-digoxigenin antibodies that were present there. Unbound PE nanoparticles traveled toward the C line, where biotin captured them, rather than reacting at the T line. Finally, the fluorescence intensities of the T line and C line were quantitatively analyzed by a rapid and portable fluorescence detection equipment (Getein Biotech, Inc., Jiangsu, China).

The probes, H1 and H2, were labeled with digoxigenin and biotin at the 5′ end, respectively. The agarose gel electrophoresis was carried out to validate the feasibility of our approach. Depending on the top-of-probe concentration ratio in previous real-time fluorescence analysis, the assay system of CHA-LFIA was further optimized. First, we identified the optimal probe concentrations by monitoring fluorescence values at different probes’ concentrations, including 100 nM, 80 nM, 60 nM, 50 nM, 40 nM, and 10 nM. Both probes and 1 nM of RNA target were mixed for 30 min at 37°C and then added to LFIA test trips. After 10 min, the fluorescence values were recorded.

The temperature and reaction time were further optimized. To determine the optimum reaction temperature, the CHA mixture was heated for 30 min at different temperatures: 25°C, 30°C, 35°C, 37°C, 40°C, 45°C, 50°C, 55°C, 60°C, and 65°C. We also evaluated the fluorescence values of LFIA for different reaction duration, including 5 min, 10 min, 15 min, 20 min, and 30 min at 37°C. Finally, we measured the fluorescence for 1 nM and 10 nM of targets in our CHA-based LFIA assay at various intervals (5 min, 10 min, 15 min, and 20 min) to identify the optimal time of fluorescence reading after adding CHA mixture on the LFIA test trip (Fig. S2). After optimizing the conditions, the sensitivity and specificity of our optimized SFTSV CHA-based LFIA assay were determined.

### Validation of CHA-based LFIA testing for SFTS RNA using clinical serum samples

All clinical samples were obtained from the affiliated Nanjing Drum Tower Hospital of Nanjing Medical University, Jiangsu, China. This clinical trial protocol was approved by Nanjing Drum Tower Hospital ethics committee (no. 2022-LCYJ-DBZ-06). The study also obtained written informed consent from the patients or legal guardians. RNA was extracted from serum of SFTS patients and healthy donors using RNA extraction kits (Daan Gene Co., Ltd, Guangzhou, China). Ten microliters of RNA samples was added to CHA probe sets for CHA-based LFIA and RT-RCR (Bio-Rad C1000 Thermal Cycler), respectively. We calculated the cut-off value for the negative fluorescence intensity using at least three assays of 12 independent negative samples. The cut-off value was designated by calculating the average value of 12 negative samples + 3 × SD. The sensitivity and specificity of SFTSV CHA-based LFIA assay were also determined using clinical samples from SFTS patients.

### Statistical analysis

GraphPad Prism 9 software was used for statistics analysis. The mean (SD) was used to present the continuous variables. Continuous variables were compared using the *t*-test or Mann–Whitney *U* test between groups. The correlation between variables was assessed using the Spearman correlation analysis. *P* < 0.05 was considered statistically significant. *P < 0.05, **P < 0.01, ***P < 0.001, ****P < 0.0001, and ns indicates no significant difference.

## RESULTS

### Principle of the CHA-based lateral flow immunoassay

**Fig 1:**
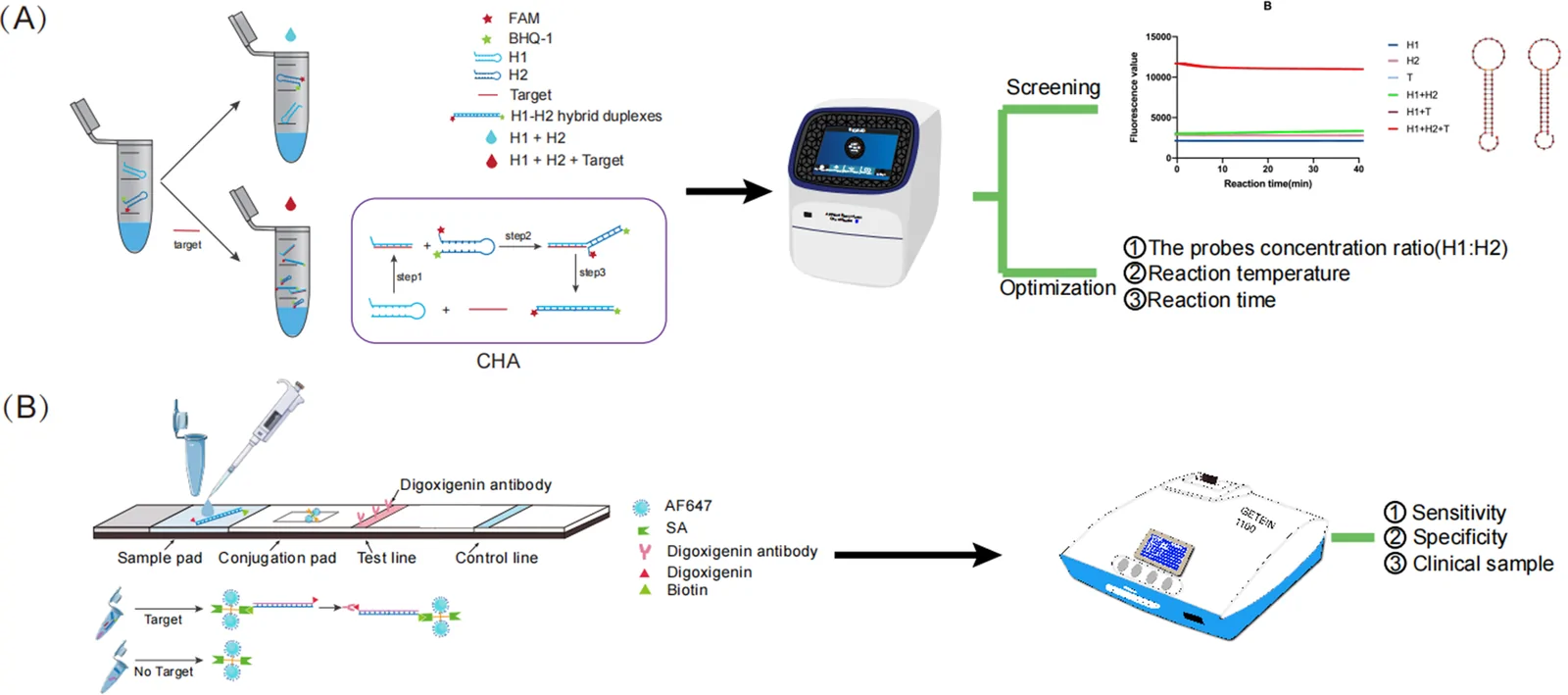
Overview of the CHA-LFIA method for SFTS viral RNA detection. (A) The study design for CHA reaction without and with addition of the target RNA. (B) The LFIA strip used to detect digoxigenin-biotin double-labeled H1-H2 hybrid duplexes. SA, streptavidin and AF647, Alexa Fluor 647.

The CHA reaction system is composed of a target strand, and a pair of complementary single-stranded hairpin DNA probes (H1 and H2) (Fig. 1A). Both probes H1 and H2 possess three contiguous zones: the stem zone, the ring zone, and the toe point zone. In the absence of targets, the two probes maintain their stable hairpin structure, as the complementary structural domains of the two probes are covered within the hairpin stem. Conversely, in the presence of RNA targets, the hairpin H1 is activated. As an enzyme catalyst or toe switch, the target RNA could facilitate the generation of H1-T complexes, which reveals a unique structural domain in the H1 stem for binding to H2 to create H1-H2 double-stranded complexes (31, 32). Signal amplification is accomplished by the reaction due to a great amount of mixed H1-H2 duplex without consuming the RNA targets. Therefore, the presence of the target RNA can be verified by detecting the H1-H2 hybrid double-stranded complex.

To further develop a simple, portable assay for SFTSV RNA testing which could be applicable in rural area or at the site of point-of-care, CHA-based amplification was further coupled with LFIA, so that we could detect the SFTSV RNA amplification signals in a portable immunofluorescence detector (Fig. 1B). To measure H1-H2 complexes by the LFIA strips, we labeled the 5′ ends of the probes for H1 and H2 with biotin and digoxigenin, respectively. The amplification products of H1-H2 hybrids double-labeled with digoxigenin-biotin were added to the LFIA strips’ sample pads. The liquid then flows to the conjugate pads. Through biotin-streptavidin interactions, the H1-H2 complexes specifically bind to the streptavidin and the fluorophore Alexa Fluor 647 double-labeled PE nanoparticles to form H1-H2 hybrids-PE nanoparticle complexes. Subsequently, the reaction solution continues to flow along the strips to the test line, where it binds specifically to the anti-digoxin antibody covering this line. The non-conjugated PE nanoparticles on the T-line move on to the C-line, in which they are captured by biotin. Finally, the fluorescence levels of the T and C lines are subsequently quantified by fluorescence detection equipment.

### Screening of SFTSV RNA hairpin probes and developing of CHA-based SFTSV RNA testing system

**Fig 2:**
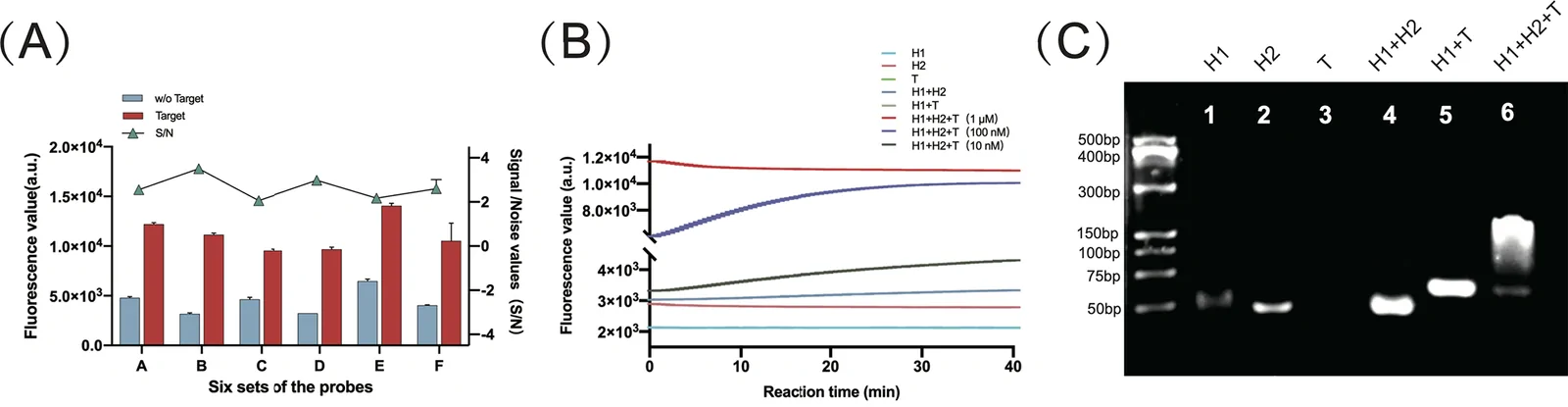
Validation of the CHA assay for detecting SFTSV RNA. (A) The fluorescence values without SFTSV RNA target (w/o target: H1 + H2) and the RNA target (H1 + H2 + T) with 1 µM of H1, H2, and the target are represented using the bar graph. The values of S/N at different groups. S/N: the fluorescence values of the fully matched RNA target to the fluorescence values without target RNA are represented by the line graph. (B) Kinetics of the CHA reaction with the B set of primers. (C) Gel electrophoresis images for the CHA reaction of the primers set of B (lane 1: H1, lane 2: H2, lane 3: target, lane 4: H1 + H2, lane 5: H1 + target, and lane 6: H1 + H2 + target). The final concentration of H1, H2, and T was 1 µM. Error bar represents the median and standard deviation for fluorescence value.

designed six sets of probes and their corresponding target RNA sequences. To monitor the CHA assay for SFTSV RNA detection, the 5′ end of the H2 was labeled with FAM fluorescence, while the 3′ end was marked with the BHQ-1 quencher group. Therefore, we were able to use real-time fluorescence analysis equipment to monitor the fluorescence levels. As a result, we found that all six probe sets and the corresponding target RNA sequences exhibited significantly higher fluorescence value compared to those without target RNA, suggesting each set of probes could successfully recognize and amplify the RNA target. The response of the probes was extremely rapid and reached plateau immediately, probably due to the high concentration of the targets (1 µM), while in the 10-nM concentration of the targets the reaction was slower and a rise in response could be observed (Fig. 2A and B; Fig. S3). Among six probe sets, the ratio of signal versus noise for probe set B was the highest (Fig. 2A). To further validate the utility of probe set B in CHA assay, AGE experiments were performed (Fig. 2C). As expected, probes H1 and H2, and the corresponding sequence formed a 102 bp H1-H2 duplex complex, suggesting the effective amplification of CHA assay. Subsequently, probe set B was selected as the ideal probe set for SFTSV RNA detection.

### Optimization of CHA fluorescence sensing systems and CHA-LFIA systems

**Fig 3:**
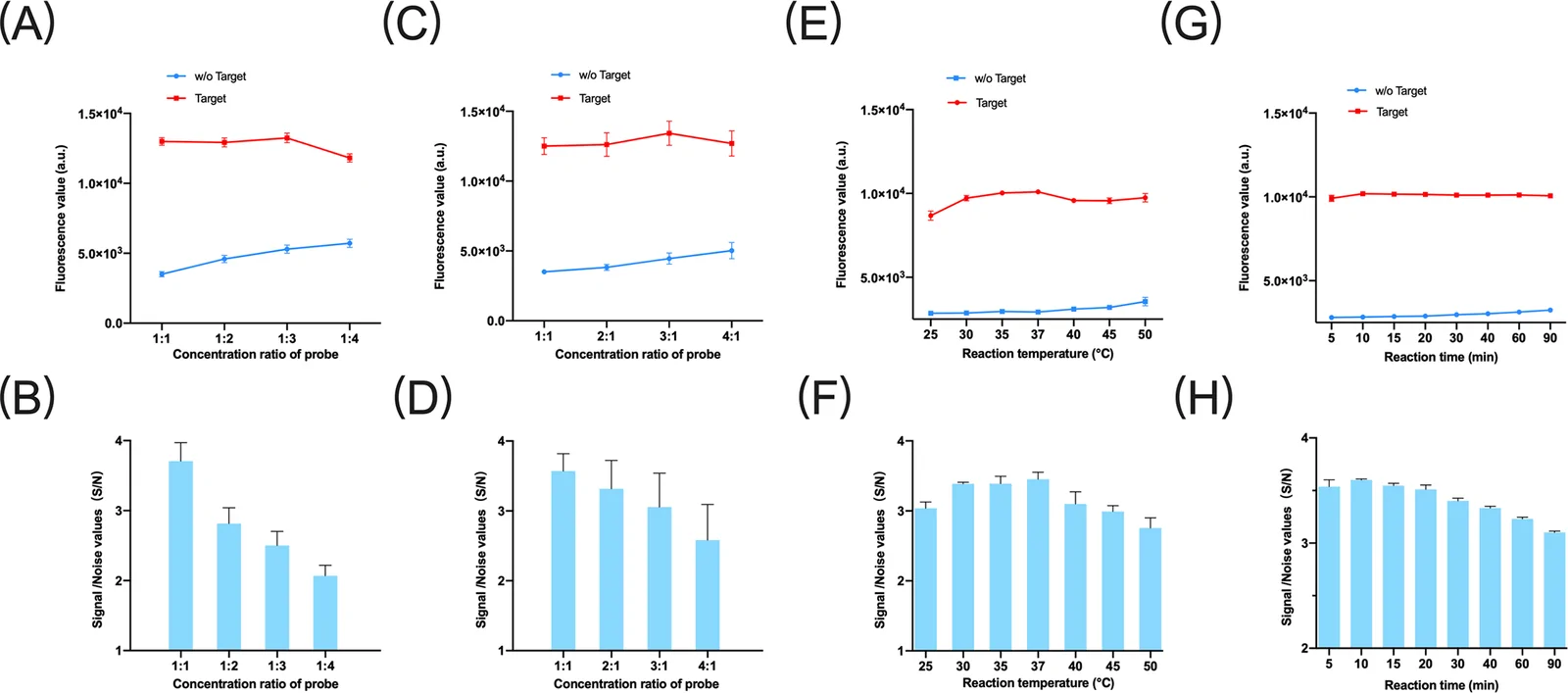
Optimization of SFTSV CHA assay. (A–D) The optimization of ratio of concentration between H1 and H2. (A) The concentration of H1 was 1 µM, and the concentrations of H2 were 1 µM, 2 µM, 3 µM, and 4 µM, respectively. (B) The values of S/N at different concentration ratios. (C) The concentration of H2 was 1 µM, and the H1 concentrations were 1 µM, 2 µM, 3 µM, and 4 µM, respectively. (D) The values of S/N at different concentration ratios. (E and F) The optimization of temperature of the CHA reaction. (E) The reaction temperature optimization using a 40-min reaction with 1 µM of H1, H2, and the target. (F) The values of S/N at different reaction temperatures. (G and H) The time of the CHA reaction. (G) The CHA reaction time at 37°C with 1 µM of H1 and H2, and 333 nM of the target. (H) The values of S/N at different reaction times. Error bar represents the median and standard deviation for fluorescence value or signal/noise values.

To achieve the best analytical performance of our SFTSV RNA testing using CHA, the reaction parameters, including DNA hairpin probes concentration ratio, probe concentration, temperature, and duration time, were optimized (Fig. 3). First, the relative signal versus noise values (S/N) were analyzed to identify the appropriate hairpin probe ratio of H1 and H2. Our results suggested that the ratio of probes H1 and H2 at 1:1 yielded the highest S/N value (Fig. 3A through D). Next, we measured the fluorescence and S/N signals at different temperatures (Fig. 3E and F) and assay durations (Fig. 3G and H). Our results suggested that CHA assay for SFTSV RNA testing performed optimally at 37°C for 10 min, achieving the highest S/N value.

**Fig 4:**
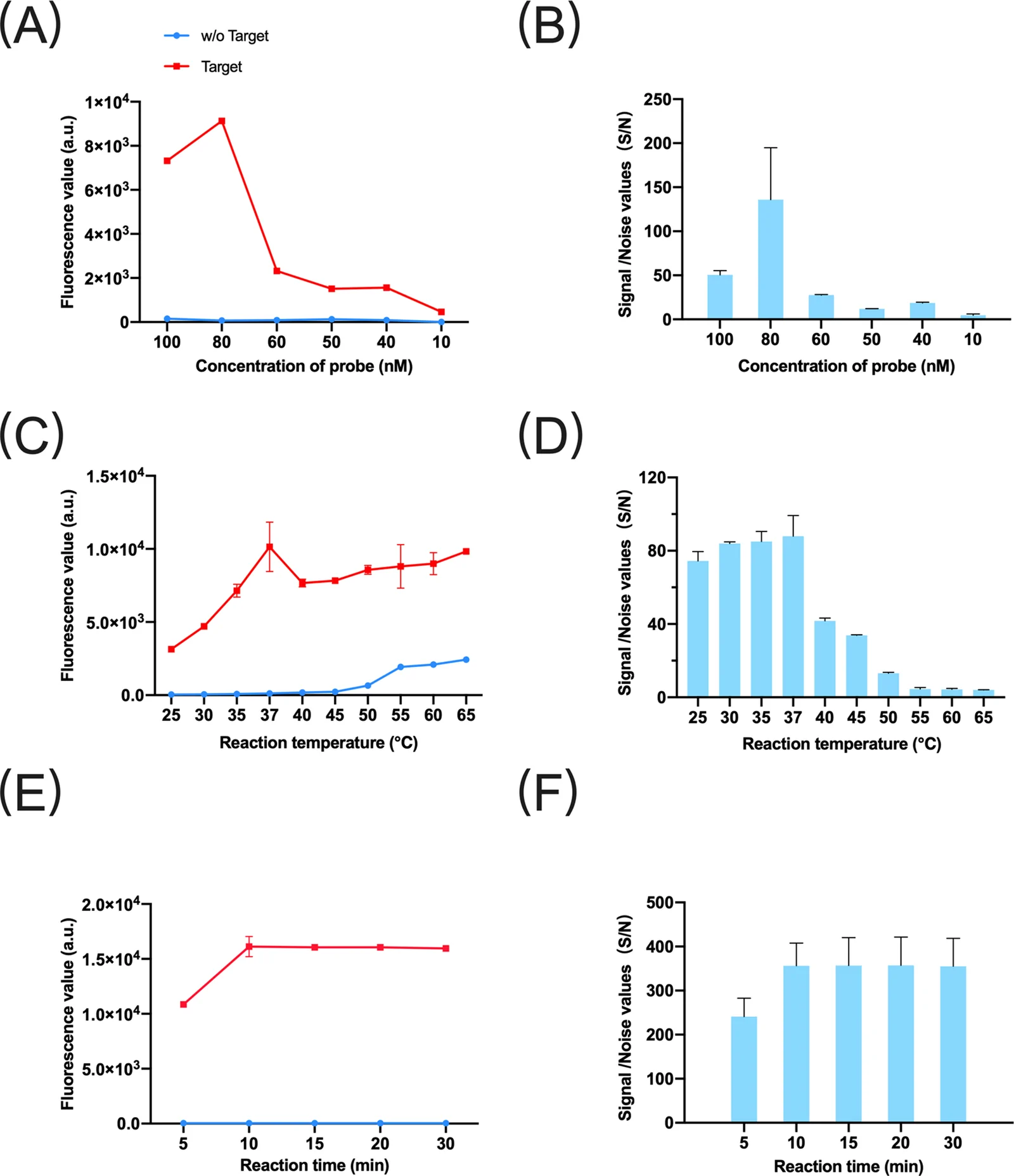
Optimization of the CHA coupled with LFIA to detect SFTSV RNA. (A) The fluorescence value of CHA coupled with LFIA at different concentrations of H1 and H2 (ranging from 10 nM to 100 nM) at a fixed ratio of 1:1. (B) The values of S/N at different probe concentrations. (C) The fluorescence value of CHA coupled with LFIA at different reaction temperatures (ranging from 25°C to 65°C) with 80 nM of H1, H2, and 1 nM of the target. (D) The values of S/N at different probes' concentration, temperature, and time, respectively. (E) The fluorescence value of CHA coupled with LFIA at different reaction times (ranging from 5 to 30 min) at 37°C with 80 nM of H1 and H2, and 10 nM of the target. (F) The values of S/N at different times. Error bar represents the median and standard deviation for fluorescence value or signal/noise values.

Given these optimized CHA assay conditions, we further aimed to determine the appropriate probe concentration, assay temperature, and testing time for the SFTSV RNA CHA-based LFIA testing system. To achieve the optimal S/N values, various concentrations of probes (the ratio of H1: H2 as 1:1) were tested, including 100 nM, 80 nM, 60 nM, 50 nM, 40 nM, and 10 nM (Fig. 4A). We identified that the probe concentration at 80 nM had the optimal S/N values (Fig. 4B). Intriguingly, the baseline fluorescence value for 100 nM of probes H1 and H2 was higher than those with relative lower probe concentration, suggesting that the high concentration of probes H1 and H2 might generate a background leak. We subsequently measured the fluorescence value under different assay temperatures (Fig. 4C and D) and the timing for fluorescence detection (Fig. 4E and F) after adding the CHA products to the LFIA test trips. We found that the assay temperature at 37°C and 10-min duration for fluorescence detection could yield the best S/N value.

### Sensitivity and specificity of the CHA-based LFIA assay

**Fig 5:**
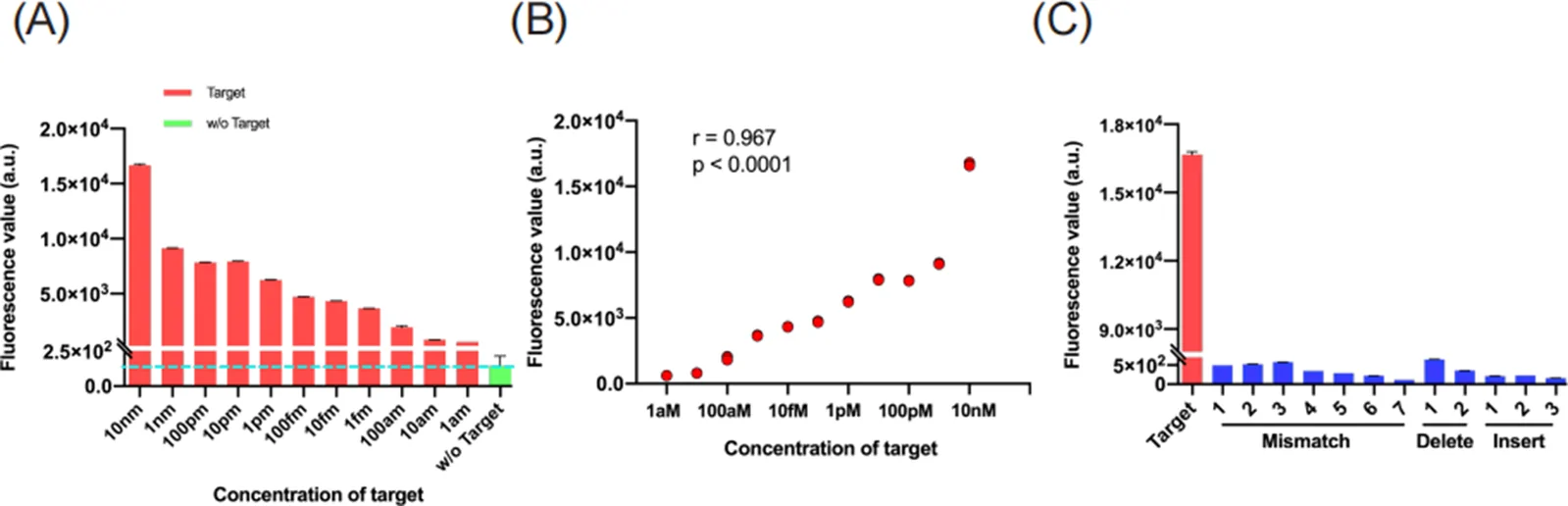
Performance of the CHA reaction. (A) Sensitivity of the CHA reaction. The fluorescence value of CHA-LFIA assay to detect SFTSV RNA target in different reaction concentrations (10 nM, 1 nM, 100 pM, 10 pM, 1 pM, 100 fM, 10 fM, 1 fM, 100 aM, 10 aM, 1 aM). (B) Correlation between the concentration of different targets and the corresponding fluorescence values. (C) The specificity of the CHA reaction, including seven mismatched sequences, two deleted sequences, and three inserted sequences. The specific sequences are listed in Table 2. These 12 non-target sequences and the RNA target were at concentrations of 10 nM. The results were obtained for three independent repeats. The dotted green line represents the blank buffer without targets. Error bar represents the median and standard deviation for fluorescence value.

To determine the limit of detection (LOD) of SFTSV RNA using our CHA-based LFIA assay under optimized assay conditions, we mixed 10-fold serial diluted target RNA sequence starting from 10 nM to 1 aM with 80 nM of H1 and H2 probes, respectively. The fluorescence measured from CHA-based LFIA assay gradually declined as the concentration of SFTSV RNA targets was serially diluted (Fig. 5A). Besides, there was a correlation between the fluorescence derived from CHA-based LFIA tests and target concentration (*R* = 0.967, *P* < 0.0001) (Fig. 5B). Additionally, the LOD of our assay to identify the presence of SFTSV target sequence was as low as 1 aM.

To further determine the specificity of our CHA-LFIA testing system, 12 non-target RNA oligonucleotides were synthesized (Table 2), including single-base, multi-base mismatched sequences, deleted sequences, and inserted sequences, as well as blank buffer. All the target or no-target RNA probes were used at a fixed concentration of 10 nM (Fig. 5C). The SFTSV RNA target exhibited high-level fluorescence values, while the mutated sequences or blank control had nearly undetectable fluorescence signals. Our results indicated that our assay could recognize SFTSV RNA sequence in a highly specific manner.

**TABLE 2:** The no-target RNA oligonucleotides tested in this study^*a*^

Name	Sequence (5′−3′)
Mismatch-1	*C*GAACAAAGAGGAG UCAACAG
Mismatch-2	*CC*AACAAAGAGGAG UCAACAG
Mismatch-3	AGAACA*G*AGAGGAG UCAACAG
Mismatch-4	AGAA*GC*AAGAGGAG UCAACAG
Mismatch-5	AGAACAAAGAG*C*AG UCAACAG
Mismatch-6	*CAGCU*A*C*AGAGCAGUCAACAG
Mismatch-7	AGAACAAAGAGGACG*A* *UCUGU*
Deletion-1	A_ AACAAAGAGGAG UCAACAG
Deletion-2	AGAACAA_GAGGAGUCAACAG
Insertion-1	A*C*GAACAAAGAGGAG UCAACAG
Insertion-2	AGA*C*ACA*C*A*U*AGAGGAGUCA*G*ACAG
Insertion-3	AGAACAA*C*AGAGGAG UCAACAG

^*a*^ Mismatch-1, -3, and -5 are single-base mismatch sequences. Other mismatch sequences are multi-base mutation sequences. Deletion, insertion-1, and -3 are single-base mutation sequences. Insertion-2 is multi-base mutation sequence. Italic indicates mismatched or inserted sequence. Blank indicates the deleted sequences.

### Detection of clinical sample from SFTS patients and healthy donors using established CHA-based LFIA assay

**Fig 6:**
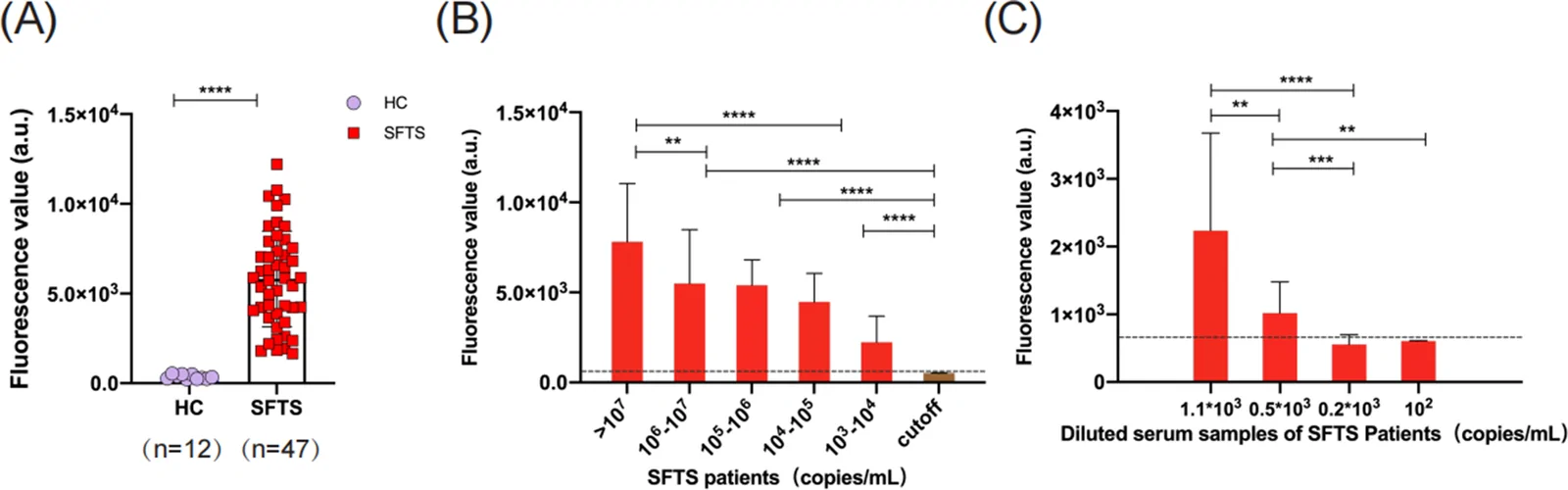
Clinical validation of CHA-LFIA assay for serum samples from SFTS patients. The fluorescence values of 47 SFTS patients and 12 health volunteers. (B) The fluorescence values of 47 SFTS patients which were further grouped in different levels of SFTSV RNA viral loads. (C) Determination of limit of detection of CHA-LFIA assay using clinical samples. Serum from SFTS patients was further diluted as 500, 200, 100 copies/mL. Error bar represents the median and standard deviation for fluorescence value. The cut-off value = the average value of 12 negative samples + 3 × SD. The dotted gray line represents the cut-off value. SFTS: SFTS patients and HC: health control. Wilcoxon un-pairs signed-rank with *P*-value was used for comparison between groups. **P < 0.01, ***P < 0.001, ****P < 0.0001, and ns: no significant difference.

To verify the applicability of our established CHA-based LFIA tests for SFTS clinical samples, we collected 47 serum samples from SFTSV patients confirmed by RT-PCR and 12 sera from healthy donors at Nanjing Drum Tower Hospital, Nanjing, China. Using our newly established CHA-based LFIA assay, fluorescence values of serum samples from SFTS patients were significantly higher than those from healthy donors (Fig. 6A). Given the levels of SFTS RNA copies revealed by RT-PCR test, we further divided the patient sera into eight groups and compared the fluorescence values among different groups (Fig. 6B). The fluorescence values derived from CHA-based LFIA assay were also compared with viral copies tested by RT-PCR results (Table 3). The fluorescence values for the serum samples from 1.1 × 10^3^ to 7.9 × 10^7^ were significantly greater than the cut-off values (*P* = 0.0006, 0.0013, 0.0007, 0.001, 0.0007), demonstrating that results between two assays were comparable. To further explore the LOD of our CHA-based LFIA assay using clinical samples, the positive samples were subjected to serial dilutions, and serum samples containing 500, 200, and 100 copies/mL of SFTS RNA were yielded. Our data showed that the fluorescence value of serum samples containing 500 copies/mL of SFTSV RNA, but not those containing 200 copies/mL and 100 copies/mL, was above the cut-off values (Fig. 6C), suggesting that LOD of our CHA-LFIA is at least as low as 500 copies/mL.

**TABLE 3:** Comparison of CHA-LFIA and RT-PCR for SFTSV RNA in 59 serum samples from SFTS patients and healthy donors

Samples	CHA-LFIA	
	Positive	Negative
RT-PCR positive (*n* = 47)	47 (100%)	0
RT-PCR negative (*n* = 12)	0	12 (100%)

## DISCUSSION

As an emerging infectious disease, SFTS has been reported in central and eastern China, Korea, and Japan (1, 33 – 35). Recently, similar cases were also reported in Vietnam (9). The global epidemiology regarding the incidence rate of SFTS over the last decade remains elusive. A meta-analysis showed that the overall pooled seroprevalence of SFTSV antibodies was 4.3%, based on 21 studies from 7 provinces in China. The pooled prevalence among farmers was 6.1% and among others it was 3.3% (36). Nevertheless, between 2010 and 2019, only 13,824 SFTS cases were reported, with a yearly escalating trend (37). It is estimated that 8.3% of SFTS cases was missed in SFTS high-endemic provinces in China (38). Since currently, only RT-PCR approach was used for clinical diagnosis of SFTS, but it is not yet widely used in rural areas, especially with limited medical resources. Nevertheless, those SFTS high-endemic provinces were mostly rural areas, and the early diagnosis and the investigation of incidence rate of SFTS cases are very difficult. Therefore, the establishment of our SFTSV CHA-LFIA is critical and is urgent to enhance our understanding of the global epidemiology of SFTS cases.

There is an increasing trend for reported SFTS infection cases in Asia (39, 40). Currently, RT-PCR is considered as the “gold standard” for SFTSV diagnosis, which relies on expensive instruments and well-trained personnel. However, due to limited medical resource in endemic rural areas, the diagnosis of SFTS patients and the appropriate treatment might be delayed, leading to a high fatality rate (37). Thus, we established a novel, simple, portable strategy for SFTSV RNA detection through integration of CHA and LFIA, which was of great importance for early detection and diagnosis of SFTSV in rural areas where ticks are prevalent. The CHA system was composed of two complementary DNA probes (H1 and H2) that were labeled with digoxigenin and biotin, respectively. In the presence of the SFTSV RNA sequence, H1 and H2 probes were constantly catalyzed to form a duplex, resulting in the exposure of digoxigenin molecules on the surface, which could be captured by LFIA strip. Taking advantage of the signal amplification of CHA and the simplicity of LFIA, we established a rapid and sensitive detection assay for SFTSV nucleic acid, which is ready to test clinical samples with minimum background noise.

CHA is considered as an efficient, highly sensitive, enzyme-free method of isothermal amplification that was widely applied in the detection of microRNA, DNA, metal ions, protein and tumor exosome (20, 29, 41, 42). CHA has been used to integrate with enzyme-linked immunosorbent assay (ELISA) techniques for SARS-CoV-2 cDNA within 2 h (43). CHA combined with surface-enhanced Raman spectroscopy could achieve the goal of simultaneously detecting multiple tumor-associated microRNAs (44). Step polymerization catalytic hairpin assembly using an electrochemical biosensor was developed to measure the serum exosomal miR-181 (45). Nevertheless, most CHA-based detecting strategies required sophisticated equipment and complex operation steps, which added to the complexity of the experiment and consequently limited their clinical applications. Compared to the mentioned CHA-based method, the simplicity of our CHA-based LFIA assay allows effective, rapid diagnosis of disease through point-of-care test (POCT) (46).

There are also some other immunoassays for SFTSV diagnosis. For example, ELISA and indirect immunofluorescence assay were developed effectively for measuring SFTSV-specific IgG and IgM antibody, respectively (47 – 50). However, the IgM antibody showed delayed reaction, with only 32% IgM positivity during day 5 to day 9 after the onset of symptoms, thus making it unavailable for early diagnosis (51). Additionally, we previously demonstrated that defective serological responses were observed in the deceased patients, which reveals the absence of specific IgG to viral nucleocapsid and glycoprotein due to the impaired differentiation of B cells and T follicular helper cells (52).

Herein, we propose a simple, sensitive, rapid, and portable assay combining CHA and LFIA strip, which was ready to apply in healthcare facilities even for rural areas with inadequate medical resources. The newly established CHA-LFIA sensing system could achieve stable detection of 500 copies/mL of SFTSV in the serum samples within a total of 30 min.

The conditions of CHA-LFIA system were optimized to achieve the optimal reaction efficiency. In this study, the maximum relative fluorescence value (S/N) was determined when probes’ concentration was 80 nM. Analysis of the optimal reaction temperature and reaction time showed that the optimal reaction efficiency could be achieved in 10 min with minimal background at 37°C. Besides, the optimal probe concentration in the CHA-based LFIA system was investigated, exhibiting a high sensitivity of LOD of 1 aM for target RNA. Moreover, our study also validated the feasibility of detecting SFTSV RNA using clinical samples from SFTS patients and healthy controls, which were consistent with the RT-PCR results.

Our study also had some limitations. First, our CHA assay coupled with detection system requires previous RNA extraction from the clinical samples. Nevertheless, RNA extraction from serum could be performed in a high throughput and automatic manner. Second, we tested a relatively small size of serum samples from SFTS patients. Additional clinical samples, especially for those longitudinal samples, could be further tested in our CHA-based LFIA system for further clinical validation.

In summary, here we established a novel strategy for SFTSV RNA detection through the integration of CHA and LFIA, which exhibits potential clinical application to facilitate the early diagnosis and treatment of SFTSV infection especially in endemic areas with limited medical resources.

## Data Availability

Data will be made available on request.

## SUPPLEMENTAL MATERIAL

Figure S1 spectrum.02144-23-s0001.pdf Fig S1. The structure of probe H1 and H2. The predicted secondary structures of DNA hairpins in various reaction temperatures (25°C, 30°C, 35°C，37°C, 40°C, 45°C, 50°C, 55°C, 60°C, 65°C) performed by NUPACK software package.

Figure S2 spectrum.02144-23-s0002.pdf Optimization of the CHA coupled with LFIA to detect SFTSV RNA. The fluorescence value of CHA coupled with LFIA at different reaction time (ranging from 5 min to 20 min) at 37°C with 80 nM of H1 and H2, and 10 nM and 1 nM of the targets.

Figure S3 spectrum.02144-23-s0003.pdf Kinetics of the CHA reaction with six sets of primers from A to F. The final concentration of H1, H2 and T was 1 μM.

Supplemental material legends spectrum.02144-23-s0004.pdf Legends of Fig. S1 to S3.

ASM does not own the copyrights to Supplemental Material that may be linked to, or accessed through, an article. The authors have granted ASM a non-exclusive, world-wide license to publish the Supplemental Material files. Please contact the corresponding author directly for reuse.
