# Supplementary material for: Rapid and portable bunyavirus SFTSV RNA testing utilizing catalytic hairpin assembly coupled with lateral flow immunoassay
Source: Microbiol Spectr. 2023 Sep 8;11(5):e02144-23. doi: 10.1128/spectrum.02144-23 (PMC10581038; doi:10.1128/spectrum.02144-23)

**Figure S1.** The predicted secondary structures of DNA hairpins in various reaction temperature (25℃，30℃，35℃，37℃，40℃，45℃，50℃，55℃，60℃，65℃) performed by NUPACK software package.

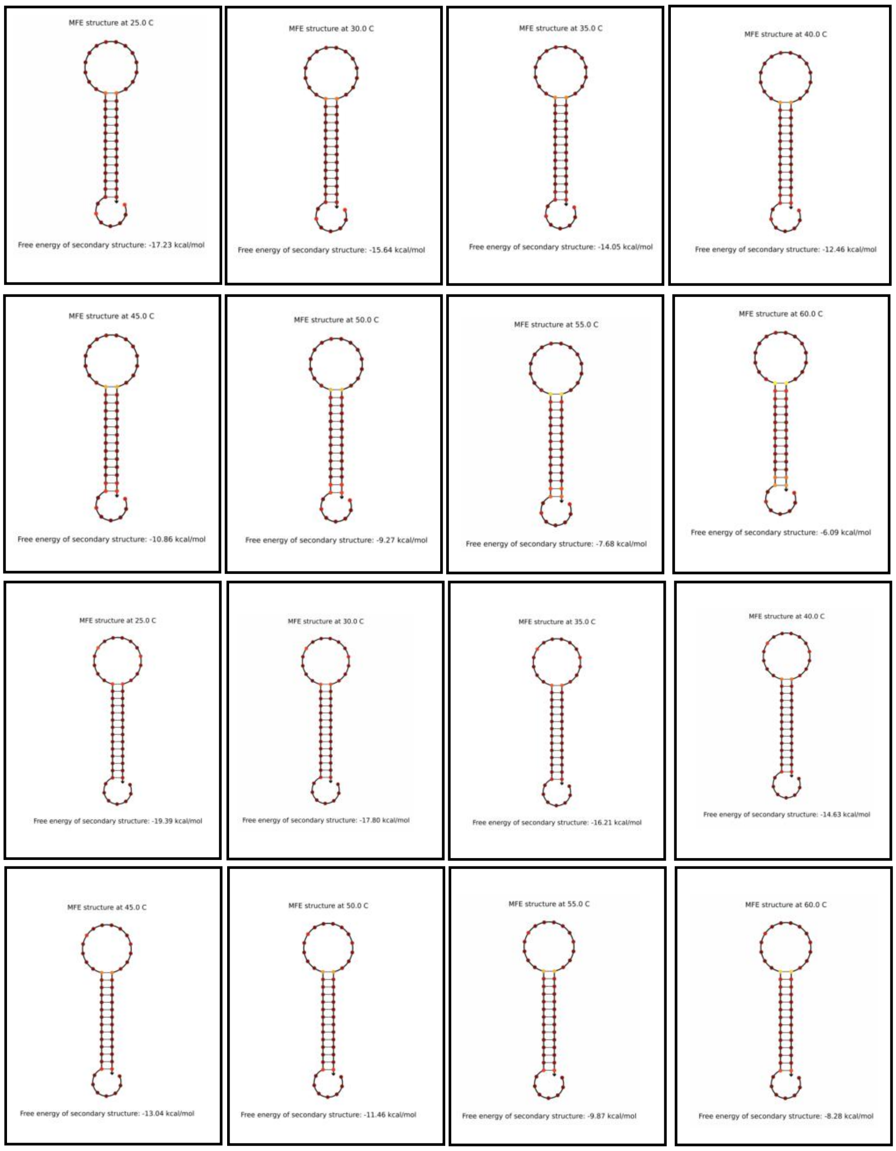

Supplement: Figure S1 — Fig S1. The structure of probe H1 and H2. The predicted secondary structures of DNA hairpins in various reaction temperatures (25°C, 30°C, 35°C，37°C, 40°C, 45°C, 50°C, 55°C, 60°C, 65°C) performed by NUPACK software package. [file spectrum.02144-23-s0001.pdf]
