# Supplementary figures and images for: Rapid and portable bunyavirus SFTSV RNA testing utilizing catalytic hairpin assembly coupled with lateral flow immunoassay
Source: Microbiol Spectr. 2023 Sep 8;11(5):e02144-23. doi: 10.1128/spectrum.02144-23 (PMC10581038; doi:10.1128/spectrum.02144-23)

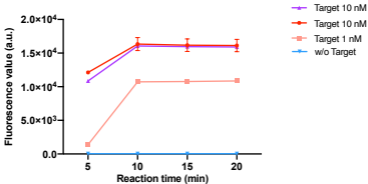

Supplement: Figure S2 — Optimization of the CHA coupled with LFIA to detect SFTSV RNA. The fluorescence value of CHA coupled with LFIA at different reaction time (ranging from 5 min to 20 min) at 37°C with 80 nM of H1 and H2, and 10 nM and 1 nM of the targets. [file spectrum.02144-23-s0002.pdf]

A

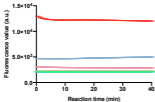

B

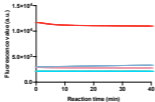

C

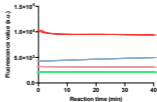

T  
H1  
H2  
H1+H2  
H1+T  
H1+H2+T

D

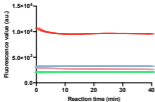

E

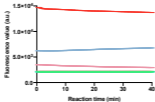

F

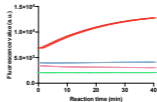

Supplement: Figure S3 — Kinetics of the CHA reaction with six sets of primers from A to F. The final concentration of H1, H2 and T was 1 μM. [file spectrum.02144-23-s0003.pdf]
