# Supplementary material for: Rapid and portable bunyavirus SFTSV RNA testing utilizing catalytic hairpin assembly coupled with lateral flow immunoassay
Source: Microbiol Spectr. 2023 Sep 8;11(5):e02144-23. doi: 10.1128/spectrum.02144-23 (PMC10581038; doi:10.1128/spectrum.02144-23)
Supplement: Supplemental material legends — Legends of Fig. S1 to S3. [file spectrum.02144-23-s0004.pdf]

Fig S1. The structure of probe H1 and H2.

The predicted secondary structures of DNA hairpins in various reaction temperature (25°C，30°C，35°C，37°C，40°C，45°C，50°C，55°C，60°C，65°C) performed by NUPACK software package.

Fig S2. Optimization of the CHA coupled with LFIA to detect SFTSV RNA.

The fluorescence value of CHA coupled with LFIA at different reaction time (ranging from 5 min to 20 min) at 37°C with 80 nM of H1 and H2, and 10 nM and 1 nM of the targets.

Fig S3. Kinetics of the CHA reaction with six sets of primers from A to F.

The final concentration of H1, H2 and T was 1 μM.
